# Effects of Aftermarket Electronic Cigarette Pods on Device Power Output and Nicotine, Carbonyl, and ROS Emissions

**DOI:** 10.1021/acs.chemrestox.3c00213

**Published:** 2023-11-30

**Authors:** Soha Talih, Nareg Karaoghlanian, Rola Salman, Elissa Hilal, Alison Patev, Ashlynn Bell, Sacha Fallah, Rachel El-Hage, Najat Aoun Saliba, Caroline Cobb, Andrew Barnes, Alan Shihadeh

**Affiliations:** † Mechanical Engineering Department, Maroun Semaan Faculty of Engineering and Architecture, American University of Beirut, Bliss Street, PO. Box 11-0236, Beirut 1107-2020, Lebanon; ‡ Center for the Study of Tobacco Products, Department of Psychology, Virginia Commonwealth University, 821 West Franklin Street, Richmond, Virginia 23284, United States; § Chemistry Department, Faculty of Arts and Sciences, American University of Beirut, Bliss Street, PO. Box 11-0236, Beirut 1107-2020, Lebanon; ∥ Department of Health Behavior and Policy, Virginia Commonwealth University, 830 E. Main St., Richmond, Virginia 23219, United States

## Abstract

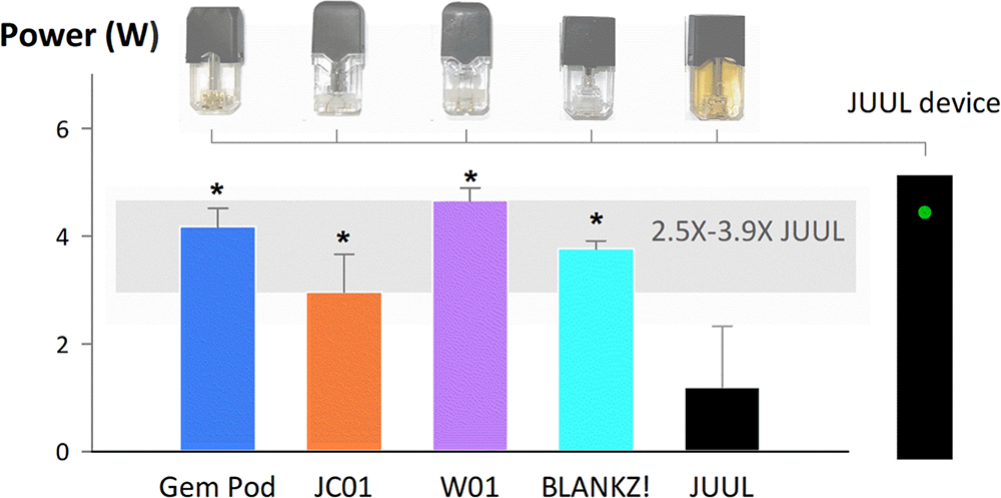

Aftermarket pods designed to operate with prevalent electronic nicotine delivery system (ENDS) products such as JUUL are marketed as low-cost alternatives that allow the use of banned flavored liquids. Subtle differences in the design or construction of aftermarket pods may intrinsically modify the performance of the ENDS device and the resulting nicotine and toxicant emissions relative to the original equipment manufacturer's product. In this study, we examined the electrical output of a JUUL battery and the aerosol emissions when four different brands of aftermarket pods filled with an analytical-grade mixture of propylene glycol, glycerol, and nicotine were attached to it and puffed by machine. The aerosol emissions examined included total particulate matter (TPM), nicotine, carbonyl compounds (CCs), and reactive oxygen species (ROS). We also compared the puff-resolved power and TPM outputs of JUUL and aftermarket pods. We found that all aftermarket pods drew significantly greater electrical power from the JUUL battery during puffing and had different electrical resistances and resistivity. In addition, unlike the case with the original pods, we found that with the aftermarket pods, the power provided by the battery did not vary greatly with flow rate or puff number, suggesting impairment of the temperature control circuitry of the JUUL device when used with the aftermarket pods. The greater power output with the aftermarket pods resulted in up to three times greater aerosol and nicotine output than the original product. ROS and CC emissions varied widely across brands. These results highlight that the use of aftermarket pods can greatly modify the performance and emissions of ENDS. Consumers and public health authorities should be made aware of the potential increase in the level of toxicant exposure when aftermarket pods are employed.

## Introduction

Consumer product regulation often has unintended consequences. One example is industry response to the FDA’s enforcement policy of February 2020 that the Agency devised to end sales of unauthorized flavored electronic cigarette cartridges other than tobacco and menthol in the USA.^1^ Cartridge-based systems are common electronic nicotine delivery systems (ENDS) that use a “pod” filled with a liquid containing nicotine and a heating coil. The pods are designed for use with a specific device that holds a battery and power circuit and are intended to be discarded when the liquid is depleted. Widely used pod systems include those marketed under the JUUL, Vuse, and NJOY brands.

Following the FDA enforcement policy, the industry began marketing off-brand refillable pods that consumers could fill with any flavored liquid. Examples of such aftermarket products include some that are compatible with the JUUL device. The JUUL device is composed of a battery, a temperature-regulating power circuit, a pressure sensor that detects a puff, and a metal case. Consumers and health authorities may assume that aftermarket pods have emissions profiles similar to those of the original manufacturer, but, to date, there are no publicly available data on the emissions of aftermarket pods. Due to variations in design and construction, these pods may modify the operation of original manufacturer devices, resulting in different nicotine and other toxicant emissions. Previous researchers found that the liquid used in prefilled aftermarket pods for JUUL contained greater numbers and concentrations of flavoring chemicals than the original pods,^2^ and that the maximum particulate yield per puff was 5.6 times higher for one aftermarket pod than JUUL.^3^

In this study, we compared the operating characteristics of the JUUL device when connected to four aftermarket pods and the original JUUL pods. We examined how the electrical power output of the device varied across pods at several puffing flow rates and how aerosol output, nicotine, and other toxicant emissions varied across products when the liquid composition was held constant. Emissions measurements included total particulate matter (TPM), nicotine, carbonyl compounds (CCs), and reactive oxygen species (ROS). CCs are thermal degradation byproducts of propylene glycol (PG) and vegetable glycerin (VG), the main constituents of ENDS liquid.^4^ CCs are associated with many pulmonary diseases in combustible cigarette smokers and include formaldehyde, a known human carcinogen.^4^ ROS initiate oxidative stress that triggers many smoking-related diseases, such as cancer.^5^

## Materials and Methods

### Materials

We used the Google search engine to identify brands of JUUL-compatible aftermarket pods available in the USA in January 2021. The search resulted in the following products: Gem Pod (UpTown Tech), W01 (OVNS TECH Co. Ltd.), JC01 (OVNS TECH Co. Ltd.), and BLANKZ! (BLANKZ! Pods). We procured nine packages of each brand from online vendors in the USA. Each package contained four pods. We also procured JUUL pods (5% nicotine, menthol-flavored) and one JUUL device. The same fully charged JUUL device was used for all measurements.

All aftermarket pods were filled with a 30/70 PG/VG (v/v), 60 mg/mL protonated nicotine solution. This formulation is consistent with previous reports on JUUL liquid composition.^6−10^ The solution was prepared using analytical-grade PG (≥99.5%, CAS 57-55-6), VG (99.0–101.0%, CAS 56-81-5), nicotine (≥99%, CAS number 54-11-5), and benzoic acid (≥99.5%, CAS 65-85-0) procured from the Sigma-Aldrich Corporation. The protonated nicotine solution was prepared by adding standard solutions of benzoic acid to freebase nicotine in a 1:1 mol ratio.

### Electrical Resistance and Resistivity

The electrical resistance of the liquid-filled pods was determined by using a standard lab Ohmmeter connected to the pod leads. Three randomly selected pods from each brand were measured in this manner. The heating coil of one pod of each brand was removed from the pod and unwound. We used a digital caliper to measure the unwound coil length and diameter. The electrical resistivity, ρ (Ωm), was determined as , where *R* is the coil resistance, *A* is the wire cross-sectional area, and *L* is the unwound coil length.

### Draw Resistance

We measured the draw resistance through each pod type at a flow rate of 1.5 standard liters per minute (SLPM). The pod was connected to a tee fitting, with one branch of the tee connected to a digital manometer (SERIES 475 Mark III Hand-held Digital Manometer, Dwyer Instruments, USA) and the other to a vacuum source regulated to produce a fixed flow rate of 1.5 SLPM with a mass flow controller (Omega FMA5400). The mouth-ends of the pods were attached to the tee fitting using a silicone sleeve that was sealed tightly around the pod. Three randomly selected JUUL pods and aftermarket pods from each brand were tested in this manner. The aftermarket pods were filled with the test liquid prior to measurement.

### JUUL Device Electrical Output

**Figure 1:**
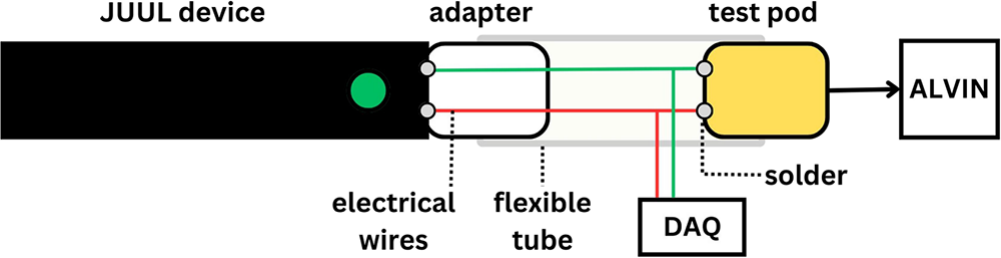
Schematic of the setup used to measure the voltage delivered by the JUUL device to each of the aftermarket and JUUL pods during puffing. ALVIN was programmed to draw ten 6 s puffs, separated by 30 s intervals, at flow rates of 1 and 2 LPM. The voltage signal delivered to the pod terminals was sampled using a DAQ. ALVIN: Aerosol Lab Vaping Instrument; DAQ: data acquisition device.

To record the JUUL device voltage output during puffing, we fabricated an adapter to provide access to the electrical output signal of the JUUL device. The adapter, which utilized a hollowed JUUL pod to interface with the JUUL device, was inserted between the device and the pods, and auxiliary electrical leads were connected to an NI USB-6001 data acquisition device (Figure 1) during and between puffs. The added electrical resistance of the adapter was approximately 4 × 10^–3^ Ω, which is negligible relative to the variability in resistance across original JUUL pods.

One pod from each brand was randomly selected and filled with liquid. The mouth-end of the test pod was connected to the American University of Beirut Aerosol Lab Vaping Instrument (ALVIN) by using a flexible sleeve to ensure a tight seal. ALVIN was programmed to generate ten puffs separated by a 30 s interpuff interval, flow rates of 1 or 2 LPM, and puff duration of 6 s, the maximum duration allowed by the JUUL device, before automatically cutting power.^11^ Additional details about ALVIN and the sampling setup can be found in Talih et al.^12^

Voltage was sampled at a rate of 20 kHz. The average voltage during each puff and across all puffs was computed for each ten-puff bout for each test pod. Power was calculated from the average voltage and the pod resistance when measured at room temperature (Table 1; we estimate the error due to this assumption to be less than 1% of the computed power).

**Table 1:** Pod Characteristics, Puff Draw Resistance, and Emissions from Four Aftermarket Pods and JUUL Pods Powered by a JUUL Device^a^

	Gem Pod	JC01	W01	BLANKZ!	JUUL
design					
inlet tube diameter (mm)	1.9	2.3	2.3	2.1	1.9
draw resistance (mm H_2_O) (*N* = 3)	166(99)	56(4.05)^b^	47(10)	98(25)	99.6(13)
wick material	silica	ceramic	cotton	silica	silica
coil surface area (mm^2^)	20	8	17	23	1
electrical characteristics					
resistivity (μΩ m)	1.01(0.09)	1.6(0.15)	1.25(0.1)	1.04(0.08)	0.98(0.1)
electrical resistance (Ω m) (*N* = 3)	2(0)^b^	1.5(0)^b^	1.8(0.01)^b^	1.4(0.01)^b^	1.6(0)
emissions in 15 puffs(*N* = 9)					
TPM (mg)	47(9.7)	59(13)	113(35)^b^	92(18)^b^	40(13)
nicotine (mg)	2.1(0.49)	2.6(0.53)	4.9(1.5)^b^	4.1(0.8)^b^	1.6(0.36)
nicotine flux (μg/s)	35(8.1)	44(8.7)	81(25)^b^	69(13)^b^	27(6)
ROS (nmol H_2_O_2_)	50(26)^b^	0.16(0.28)	58 (60)^b^	6.8 (2.5)	3.4(1.3)
carbonyls (μg)					
formaldehyde	2.8(1.4)	1.6(0.59)	9.5(4.9)^b^	0.32(0.16)	0.33(0.2)
acetaldehyde	13(8.8)	4.7 (0.41)	36(23)^b^	2.6(1.14)	4.1(1.5)
acetone	4.8(0.36)	4.6(0.1)	7.1(2.6)^b^	5.5(2)	4.1(0.21)
acrolein	0.07(0.035)	0.05(0.03)	0.14(0.067)^b^	0.03(0.05)	0.059(0.073)
propionaldehyde	1.2(1.03)	0.3(0.05)	2.4(1.4)^b^	0.28(0.15)	0.2(0.095)
crotonaldehyde	2.6(1.7)^b^	0.42(0.06)	2.8(1.6)^b^	0.48(0.22)	0.28(0.02)
methacrolein	0.84(0.014)	0.8(0.01)	1.1(0.42)	0.97(0.37)	0.86(0.043)
butyraldehyde	0.78(0.095)	0.8(0.03)	0.79(0.37)	0.5(0.21)	0.79(0.24)
valeraldehyde	ND	ND	0.06(0.19)	0.16(0.17)	0.04(0.06)
glyoxal	0.67(0.13)	0.7(0.1)	1.3(0.72)	0.45(0.19)	0.44(0.027)
methyl glyoxal	11(7.3)	3.6(1.2)	30(21)^b^	1.7(0.815)	1.05(0.3)
total CCs	40(22)	18(2.8)	113(72)^b^	13 (5.4)	12(1.8)
total CCs/TPM (μg/mg)	0.92(0.55)	0.32(0.12)	1.03(0.8)^b^	0.14(0.05)	0.35(0.15)

a Draw resistance was measured at 1.5 LPM. Emissions were measured at 1.5 LPM, 4 s puff duration, and 10 s interpuff intervals. Resistivity uncertainty values shown are based on the standard propagation of measurement uncertainty. Values are reported as mean(standard deviation).

b Indicates significant difference relative to JUUL pod.

### Puff-by-Puff Liquid Consumption

We measured the amount of liquid consumed puff-by-puff from the JUUL and aftermarket pods as described by Salman et al.^13^ Each pod was used to generate three puffing bouts consisting of ten 4 s puffs separated by a 30 s interpuff interval and flow rates of 1 and 2 LPM. During each of the 30 s interpuff intervals, the pod and battery were removed and weighed as one unit in an analytical balance. The mass of liquid consumed was computed as the difference between the pre and postpuff mass of the unit for each puff. Three new pods of each product were randomly selected and tested in this manner.

We note that for these tests, a flow rate of 1 LPM was not always sufficient to trigger the activation of the Gem Pod, but that the pod reliably fired at flow rates of 1.5 LPM and greater. Therefore, we tested the Gem Pod at 1.5 LPM (vs 1 LPM) for the lower flow rate condition. We suspect that the larger draw resistance of the Gem Pod made it more susceptible to leaks at the juncture between the pod and the JUUL device, causing some of the airflow into the pod to bypass the internal pressure sensor of the JUUL device.

### Aerosol Emissions Testing

For these measurements, each pod was attached directly to the JUUL device and connected to ALVIN via a flexible tube that was sealed tightly around the pod. ALVIN was programmed to draw 15 puffs of 4 s duration, a 10 s interpuff interval, and 1.5 LPM flow rate. These parameters are consistent with recently reported JUUL topography parameters.^14^ All pods were primed by generating three puffs of 4 s duration, 1.5 LPM flow rate, and 10 s interpuff interval prior to commencing sampling. A one-h rest period between priming and sampling sessions was provided to bring the pod back to ambient temperature.

The aerosol exiting the pod was split into two parallel flow streams, with each stream drawn through a 47 mm quartz filter (Pall Type A/E) for nicotine and ROS determination, respectively. One filter was followed by a 2,4-dinitrophenylhydrazine (DNPH) cartridge (Sigma-Aldrich/LpDNPH H10) to trap gas-phase carbonyl species. One pod from each of the nine procured packages was randomly selected, resulting in nine repeated measurements for each brand.

### Chemical Analysis

The amount of liquid consumed was determined by weighing the device and pod pre and postsampling. TPM was determined by weighing the filter pads pre and postsampling. Nicotine was measured by extracting the filter pads in 6 mL of ethyl acetate for 30 min at ambient temperature and analyzing an aliquot of the resulting solution using GC-MS, as described in the reference.^15^ The limits of detection and quantification for nicotine using this method were 0.046 and 0.153 μg/mL.

CCs were trapped, derivatized on the DNPH cartridges, and eluted with 90/10 (v/v) ethanol/acetonitrile, and quantified by high-performance liquid chromatography ultraviolet (HPLC-UV), as described by El-Hellani et al.^16^ The species analyzed and the limits of detection and quantitation were as follows (μg): formaldehyde, 0.006 and 0.019; acetaldehyde, 0.004 and 0.012; acetone, 0.002 and 0.006; acrolein, 0.002 and 0.006; propionaldehyde, 0.004 and 0.014; benzaldehyde, 0.004 and 0.013; valeraldehyde, 0.002 and 0.006; glyoxal, 0.005 and 0.018; and methyl glyoxal, 0.002 and 0.008.

ROS in the particle phase was determined using the fluorescence-based technique described by Haddad et al.^17^ In brief, 10 mL of a 2′,7′-dichlorofluorescein diacetate solution was deacetylated using sodium hydroxide (NaOH; 40 mL of 0.01 M), after which the pH was adjusted to 7.2 using a phosphate buffer solution (200 mL of 0.25 mM). Horseradish peroxidase (0.5 U/mL) was added to amplify the fluorescence signal. Fluorescence was measured using a SpectraMax M5 microplate reader against a calibration curve of H_2_O_2_ (1 × 10^–7^–10^–6^ M).

### Statistical Analysis

Outcome measures, including puff draw resistance, electrical power, TPM, nicotine, ROS, and CCs, were summarized as mean(SD). One-way analysis of variance, including posthoc pairwise comparisons (Tukey’s HSD), was used to compare outcome measures. The associations between average electrical power and the amount of liquid consumed with flow rate (1–2 LPM) and puff number (1–10 puffs) were analyzed using a general linear regression analysis. In this analysis, the β estimate represents the magnitude of change in the outcome variables (either power or liquid consumption) for a one-unit increase in the predictor (either flow rate or puff number). A significant β estimate indicates a significant effect of the predictor on the outcome. A linear regression model was used to examine the relationships between power and toxicant emissions (TPM, nicotine, total CCs, and ROS). Statistical analysis was performed using IBM SPSS version 29.0 (IBM, Armonk, NY, USA). Statistical significance was set at *p* < 0.05.

## Results

### Device Characteristics and Puff Draw Resistance

**Figure 2:**
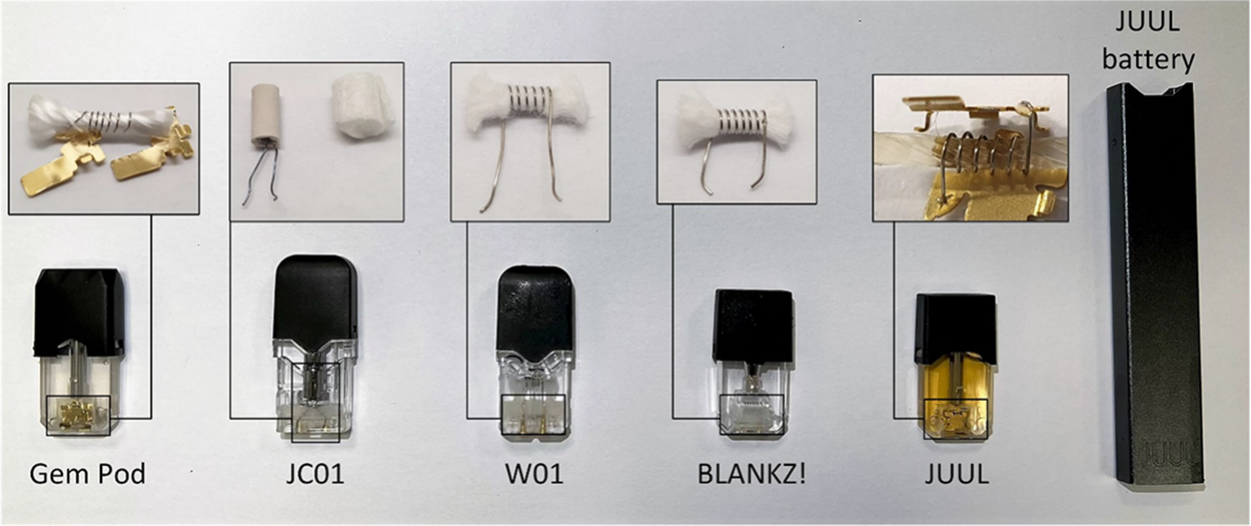
Photos of aftermarket and original JUUL pods with disassembled coil/wick systems and a JUUL device.

Photos of the pods are shown in Figure 2. All aftermarket pods fit onto the JUUL device and made a “click” sound when inserted. Apart from JC01, all models had a single horizontal heating coil wrapped around a silica wick (Gem Pod and BLANKZ!) or a cotton wick (W01). JC01 had a vertical heating coil encased in a ceramic cylinder that was covered by a textile sheath. The coil surface area ranged between 8 and 23 mm^2^. The electrical resistances varied between 1.4 and 2 Ω; all aftermarket pod resistances were significantly different from those of JUUL (1.6 Ω). The computed electrical resistivity varied between 1 and 1.6 μΩ m (nichrome: 1–1.5 μΩ m^18^). Puff draw resistance ranged between 56 and 166 mm H_2_O. We note that some of the aftermarket pods exhibited wide within-pod puff draw resistance variability. For example, Gem Pod exhibited a puff draw resistance ranging from 74 to 270 mm H_2_O. Although all tested pods clicked when inserted, the inserted Gem pods left visible gaps between the pod and JUUL device, which likely led to the observed variations in puff draw resistance. Table 1 provides a summary of the results.

### Puff-by-Puff Power and Liquid Vaporized

We found significant associations between the power and liquid vaporized across and within products. Average power for aftermarket pods ranged between 2.7 and 4.6W, all significantly greater than OEM JUUL pods (1.2–1.5W) (Table 2). The computed mean thermal efficiency of the pods, defined as the ratio of the electrical energy delivered to the pod in 10 puffs and the enthalpy required to vaporize the liquid, is also shown in Table 2. The OEM JUUL pods exhibited an efficiency of 1.5–2.5 times greater than the aftermarket pods.

**Table 2:** Liquid Vaporized (mg/puff) and Power (W) Averaged Across 10 Puffs^a^

	flow (SLPM)	power (W)	liquid vaporized (mg)	energy drawn (J)	enthalpy delivered (J)	thermal efficiency (%)
Gem Pod	1.5	4.2(0.34)^b^	4.4(0.99)	170	4.3	3
	2	4.3(0.11)^b^	4.3 (1.0)	170	4.2	2
JC01	1	3.0(0.7)^b^	2.2(0.78)^b^	120	2.2	2
	2	2.8(0.2)^b^	2.5(0.77)^b^	110	2.4	2
W01	1	4.7(0.24)^b^	7.7 (0.4)^b^	190	7.5	4
	2	4.6(0.17)^b^	8.1(0.22)^b^	180	7.9	4
BLANKZ!	1	3.8(0.15)^b^	7.5 (1.1)^b^	150	7.3	5
	2	3.7(0.1)^b^	7.5 (0.86)^b^	150	7.3	5
JUUL	1	1.2(1.1)	3.4 (2.2)	48	3.3	7
	2	1.5(0.81)	4.4(2.1)	60	4.3	7

a Liquid consumed was measured at 1 and 2 SLPM, 4 s puff duration, and 30 s interpuff intervals. Power was measured at 1 and 2 SLPM, 6 s puff duration and 30 s interpuff intervals.

b Indicates significant difference with JUUL relative to either flow rate.

**Figure 3:**
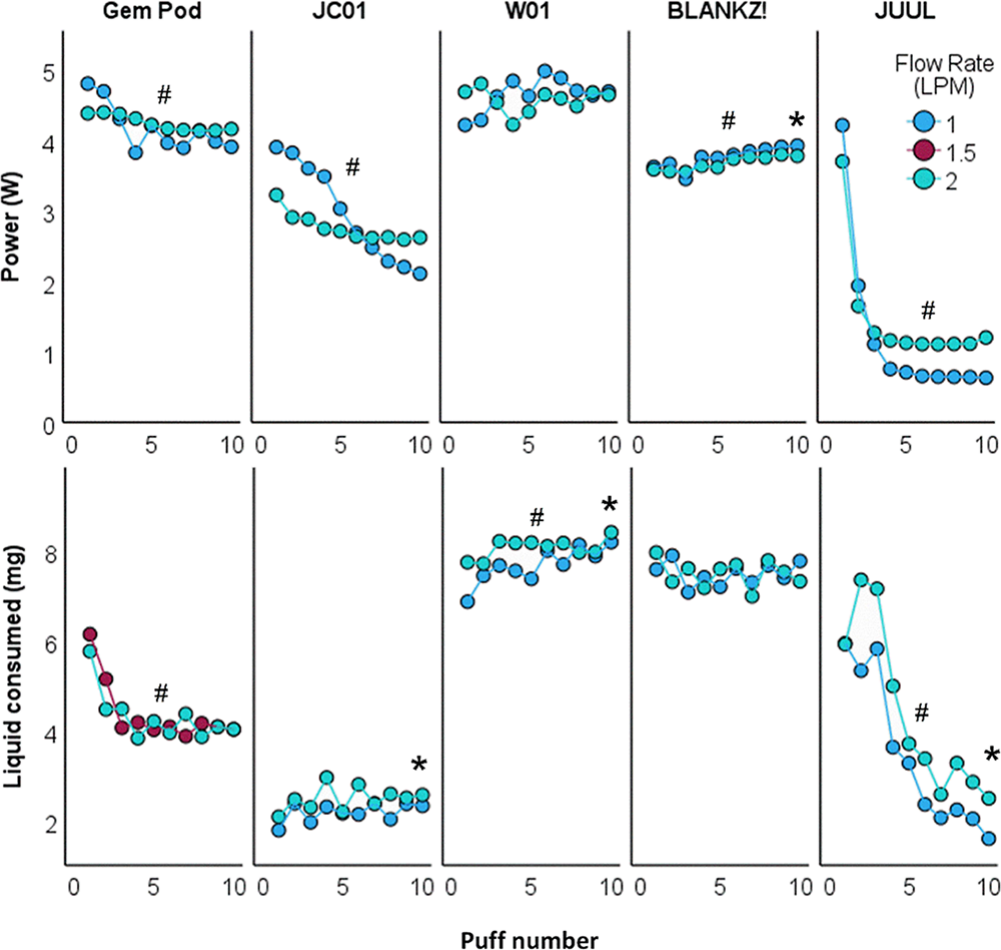
Top: effects of the flow rate (1–2 LPM) and puff number (1–10) on the average power per puff. One pod from each model was used to generate ten consecutive 6 s puffs with a 30 s interpuff interval. Each circle represents the average power per puff. Bottom: effects of the flow rate (1–2 LPM) and puff number (1–10) on the average amount of liquid consumed. Each circle represents the average amount of liquid consumed per puff obtained for the three new pods from each model. Each pod was used to generate three bouts of 10 consecutive 4 s puffs with 30 s interpuff intervals (*N* = 9 per data point). *represents significant associations between flow rate with electrical power and the amount of liquid consumed; #represents significant associations between puff number with electrical power and the amount of liquid consumed.

As shown in Table 3 and Figure 3, the influence of puff number on power and liquid consumption varied across pods. Notably, both the Gem Pod and OEM JUUL pods exhibited significant reductions in power and liquid consumption with increasing puff number. In contrast, the effects of puff number on power and liquid consumption were not consistent for JC01, W01, and BLANKZ!. The influence of the flow rate on power and liquid consumption also varied across the evaluated pods. Increasing the flow rate from 1 to 2 LPM resulted in significantly increased liquid vaporized for JUUL and two other aftermarket pods: JC01 and W01, albeit to a lower extent than JUUL (Table 3, Figure 3).

**Table 3:** Associations between Puff Number (1–10 Puffs) and Flow Rate (1–2 LPM) with Average Electrical Power and Amount of Liquid Consumed for Four Refillable Aftermarket Pods and JUUL Pods Powered by a JUUL Device^a^

outcome/predictors	metric	Gem Pod	JC01	W01	BLANKZ!	JUUL
outcome: power (W)						
predictors	puff number	–0.06(0.01); **< 0.001**	–0.1(0.02); **< 0.001**	0.02(0.02); 0.2	0.04(0.005); **< 0.001**	–0.2(0.06); **0.002**
	flow rate	0.07(0.08); 0.4	–0.2(0.1); 0.1	–0.08(0.09); 0.4	–0.08(0.03); **0.01**	0.3(0.3); 0.5
outcome: liquid consumed (mg)						
predictors	puff number	–0.1(0.04)**; 0.002**	0.03(0.02); 0.09	0.08(0.02); **< 0.001**	–0.008(0.02); 0.7	–0.5(0.06); **< 0.001**
	flow rate	–0.1(0.4); 0.7	0.3(0.1); **0.008**	0.4(0.1); **0.002**	0.004(0.1); 0.9	0.9(0.3); **0.01**

a Significant associations are shown in bold. Values are reported as β estimate(standard error); p-value; significance: *p* <.05.

### Toxicant Emissions

TPM for the aftermarket pods ranged between 46 and 112 mg, and nicotine yield ranged between 2 and 5 mg (JUUL: 40 mg TPM, 1.6 mg nicotine). Gem Pod and JC01 emitted similar TPM and nicotine yield as JUUL pods, while W01 and BLANKZ! emitted significantly greater TPM and nicotine than JUUL. ROS ranged between 0.16 and 58 nmol H_2_O_2_ (JUUL: 3.4 nmol H_2_O_2_), with Gem Pod and W01 generating significantly greater levels than JUUL. Similarly, total CC emissions ranged between 13 and 118 μg (JUUL: 12 μg); Gem Pod and W01 generated significantly higher total CCs than JUUL (Table 1). We note that two W01 pods generated CC emissions that were above the limit of quantification and were thus replaced by the upper limit of quantification for analysis. The results also show that electrical power is a significant predictor of TPM (*r* = 0.56, *p* < 0.0001), ROS (*r* = 0.49, *p* < 0.001), and total CCs (*r* = 0.53, *p* < 0.001) across pods (Figure S2).

## Discussion

This study was conducted to learn how aftermarket pods may modify the performance and toxicant emissions of ENDS relative to when original equipment manufacturer components are used. We procured four brands of refillable aftermarket pods that are marketed for use with JUUL and examined how the electrical power provided by the same JUUL device varied when it was used to power the various aftermarket pod brands and the original JUUL pods. We found that when coupled to the aftermarket brands, the JUUL device provided two to fourfold greater electrical power, resulting in up to three times the nicotine and up to fifteen times the ROS emissions as when the device was coupled to the original JUUL pods. Furthermore, our analysis revealed a significant relationship between power output and toxicant emissions, indicating that increased electrical power output contributed to elevated toxicant emissions across all pod types. Additionally, we observed significant design and construction differences across aftermarket pods.

The greater power and emissions observed with the aftermarket pods are likely the result of impairment of the JUUL device temperature control function when driving these pods. Evidence for temperature control dysfunction comes from the observation that, when coupled to aftermarket pods, the JUUL device power output did not vary substantially with the air flow rate. In principle, the greater the air flow passing through the pod and over the heated pod coil, the more the heat is lost to the air and the greater the electrical power input needed to maintain a given set temperature. Under the flow conditions prevalent in the devices studied, maintaining the coil temperature while doubling the flow rate from 1 to 2 LPM would have required approximately 1.4 times greater electrical power under ideal conditions.^15^ In reality, extraneous heat losses would dictate a greater incremental power demand factor than 1.4. However, as shown in Figure 3, doubling the flow rate from 1 to 2 LPM did not increase the power demand with any of the aftermarket pods, indicating that power was not effectively modulated to regulate temperature. In contrast, the JUUL device running at 2 LPM drew a mean power increase by a factor of approximately 1.6 (*p* < 0.0001) in puffs 3–10.

In previous reports examining OEM JUUL nicotine emissions, the effect of flow rate was not considered.^6,10,19^ However, in this study we observed a 30% rise in liquid vaporization (Table 2) when the OEM JUUL pods were operated at the 2 SLPM flow rate, an increase consistent with the greater power delivery at this higher flow. The current results suggest that previous estimates of JUUL nicotine flux and yield that utilized a 1 SLPM flow rate^10,11^ might have underestimated JUUL nicotine emissions and should be re-examined if user topography data with these products is substantially different than 1 SLPM. More generally, this study highlights the importance of explicitly addressing flow rate in testing protocols with temperature-regulated devices (vs power-regulated devices, for which emissions are insensitive to flow rate^15,20^).

We note that temperature control in ENDS devices relies on sensing the variation of electrical resistance with temperature in the heating coil; for a metal conductor, the relative change in resistance per unit change in temperature is a function of the chemical composition of the metal, as characterized by the so-called temperature coefficient of resistance (TCR). In general, as the temperature increases, so does the resistance of the coil. Because the JUUL device assumes a value of TCR that corresponds to the composition of the JUUL heating coil, any variation between the OEM JUUL coil TCR and that of an aftermarket coil will cause the JUUL temperature control circuit to misinterpret the sensed resistance. In our study, we found marked differences in intrinsic resistivity (resistance per length per cross-sectional wire area, a material property) of the JC01 and W01 aftermarket coils relative to that of the standard JUUL pods. This difference indicates that the coils in these aftermarket pods have a different composition than the OEM JUUL pods and therefore very likely a different TCR. An aftermarket pod with a lower TCR than the OEM JUUL would cause the device to underestimate the coil temperature, leading it to continue to supply power even when the coil has exceeded the set point. This is a potential issue with aftermarket JUUL pods. The resulting higher power with the aftermarket JUUL pods, in turn, results in an increase in TPM, total CCs, and ROS (Figure S2). We note that while power predicts toxicant emissions, temperature differences may also contribute to increased levels of toxicants in certain cases. For example, the difference in total CCs per unit of TPM between OEM JUUL and W01 pods (Table 1) suggests that its higher emissions not only result from higher power but also higher temperature.

In addition to potential differences in TCR across pods, the wick design plays a pivotal role in emissions. Our earlier research showed that JUUL devices paired with “new technology” JUUL pods featuring a cotton wick instead of the previous generation’s silica wick consistently maintained a higher voltage to the heating coil.^23^ The greater voltage resulted in a 50% increase in nicotine and particulate emissions. In the current study, both JC01 and W01 pods used wick materials different from those of JUUL, which might have influenced emissions.

As previously reported for JUUL products,^13^ the first puffs involve significantly greater per-puff emissions. In this study, we found that not only OEM JUUL pods but also Gem Pods emit more TPM during the first puffs. The decrease in emissions with puff number can be attributed to the formation of air bubbles around the wick.^21^ The buildup of bubbles can prevent full wetting of the coil by the liquid, leading to decreased emissions. The emissions return to normal when the bubbles are detached from the wick by flicking, removing and reinstalling, or squeezing the pod.^21,22^

We also observed that toxicant emissions from aftermarket pods generally exhibited higher variability than JUUL pods (Table 1). This variability was the most pronounced for W01 (RSD: 57% for ROS compared to 14% for JUUL and 104% for CCs compared to 38% for JUUL), and we suspect it stems from looser manufacturing tolerances. We have previously found that manufacturing variations in nominally identical products can significantly influence CC emissions.^24^ In addition, well-functioning temperature control circuitry might reduce toxicant emission variability, even where there are manufacturing variations.^24^

Limitations of this study include the use of a low 10 s interpuff interval when generating aerosols for measuring toxicant emissions, and the aftermarket pods were filled with a liquid composed only of PG, VG, nicotine, and benzoic acid. While they would be very unlikely to affect the gross mechanical behavior of the liquid,^25^ trace additives used in original JUUL pods might have increased the toxicity profile of the aftermarket pods.^26,27^ In addition, for convenience, the study employed an intensive puffing regimen for emissions testing. In particular, a 10 s interpuff interval is shorter than typically found in human participant studies. Nonetheless, the same puffing protocol was used for all products in this study, enabling a comparison on the same basis.

We have previously argued that only closed-system ENDS can be regulated effectively for nicotine emissions because closed systems do not allow users to modify factors such as liquid and power,^28^ and perhaps prematurely invoked JUUL as an example of a closed system. In this study, we found that users can effectively dial up the power of the JUUL device by pairing it with some aftermarket pods and, in the process, attain greater nicotine and other toxicant fluxes. In effect, aftermarket manufacturers have reopened what would otherwise have been considered “closed” systems.

The findings of this study highlight the need to inform consumers that aftermarket pods may impair the temperature control circuitry of OEM devices, potentially leading to greater exposure to harmful chemicals. They also highlight the need for regulators to develop stronger models for predicting industry responses to regulations, thereby reducing unintended consequences.

## Supporting Information Available

Photo of the flexible tubing attached to the pod and relationship between power and toxicant emissions (TPM, ROS, and total CCs) (PDF)

## Abbreviations

ALVIN: American University of Beirut Aerosol Lab Vaping Instrument
CCs: carbonyl compounds
DNPH: 2,4-dinitrophenylhydrazine
DAQ: data acquisition system
ENDS: electronic nicotine delivery system
FDA: Food and Drug Administration
HPLC-UV: high-performance liquid chromatography with ultraviolet
NIH: National Institute of Health
OEM: original equipment manufacturer
PG: propylene glycol
ROS: reactive oxygen species
SD: standard deviation
TCR: temperature coefficient of resistance
TPM: total particulate matter
VG: vegetable glycerin
