# Supplementary material for: Effects of Aftermarket Electronic Cigarette Pods on Device Power Output and Nicotine, Carbonyl, and ROS Emissions
Source: Chem Res Toxicol. 2023 Nov 30;36(12):1930–7. doi: 10.1021/acs.chemrestox.3c00213 (PMC10731641; doi:10.1021/acs.chemrestox.3c00213)
Supplement: Supplementary file 1 — tx3c00213_si_001.pdf [file tx3c00213_si_001.pdf]

Supporting Information

Effects of aftermarket electronic cigarette pods on device power output and nicotine, carbonyl, and ROS emissions

Soha Talih[†, ‡], Nareg Karaoghlanian[†, ‡], Rola Salman[†, ‡], Elissa Hilal[†], Alison Patev[‡], Ashlynn Bell[‡], Sacha Fallah[§, ‡], Rachel El-Hage[§, ‡], Najat Aoun Saliba[§, ‡], Caroline Cobb[‡], Andrew Barnes[‡, ‖], Alan Shihadeh[†, ‡]*

† Mechanical Engineering Department, Maroun Semaan Faculty of Engineering and Architecture, American University of Beirut, Bliss Street, PO. Box 11-0236, Beirut, Lebanon
‡ Center for the Study of Tobacco Products, Department of Psychology, Virginia Commonwealth University, 821 West Franklin Street, Richmond, Virginia 23284, United States
§ Chemistry Department, Faculty of Arts and Sciences, American University of Beirut, Bliss Street, PO. Box 11-0236, Beirut, Lebanon
‖ Department of Health Behavior and Policy, Virginia Commonwealth University, 830 E. Main St., Richmond, VA, 23219, United States

Table of Contents

Figure S1. Photo of the flexible tubing attached to the pod.

Figure S2. Relationship between power and toxicant emissions (TPM, ROS, and total CCs). The lines represent linear regression models showing the association between power and the specific toxicants analyzed.

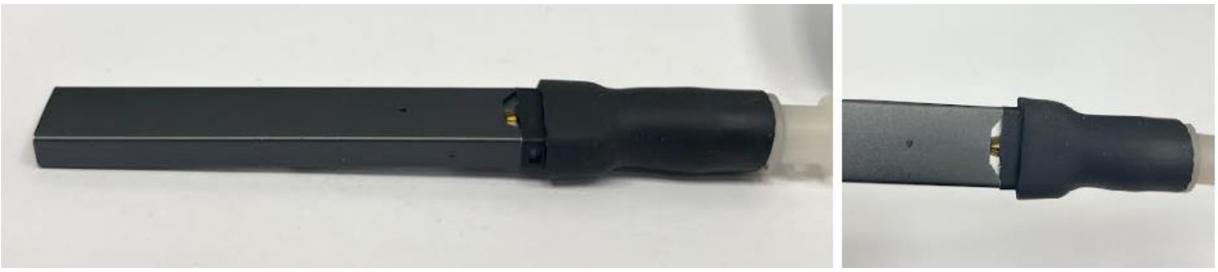

Figure S1. Photo of the flexible tubing attached to the pod.

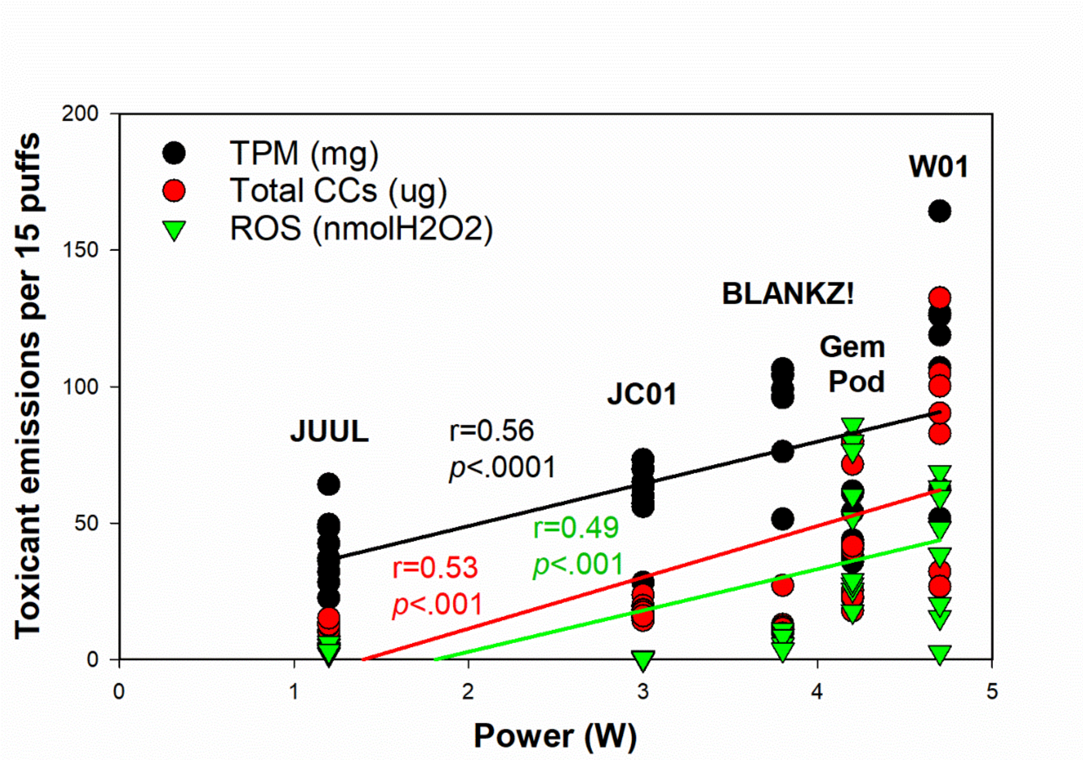

Figure S2. Relationship between power and toxicant emissions (TPM, ROS, and total CCs). The lines represent linear regression models showing the association between power and the specific toxicants analyzed.
